# Materials analysis and image-based modelling of transmissibility and strain behaviour in approved face mask microstructures

**DOI:** 10.1038/s41598-022-22102-6

**Published:** 2022-10-17

**Authors:** Manoochehr Rasekh, Francesca Pisapia, Ashley Howkins, David Rees

**Affiliations:** 1 College of Engineering, Design and Physical Sciences, Brunel University London, Uxbridge, UB8 3PH UK; 2 Newcells Biotech, The Biosphere, Drayman Helix, South St, Newcastle upon Tyne, NE4 5BX UK

**Keywords:** Biomedical engineering, Materials science

## Abstract

Comparisons are made between six different approved face masks concerning their particle transmissibility allied to mechanical properties. The latter involves material testing and stretch or strain behaviour under load. SEM and X-ray elemental analyses showed contrasting structures between random and ordered fibre orientations. These constitute the mask designs where transmissibility is to be minimised. Airflow velocity measurement enabled filtration to be measured between the different mask designs, from two to six layers of different fabrics in combination. SEM provided the fibre diameter and pore size of each mask layer, up to a maximum of six. Stretching each complete mask showed its elasticity and recovery behaviour on an energy basis. The energy conversion involved in mask straining involves areas enclosed within steady and cyclic load-extension plots. Thus, the work done in extending a mask and the energy recovered from its release identified a hysteresis associated with an irrecoverable permanent stretch to the mask fabric. Failure of individual layers, which occurred successively in extended stretch tests, appeared as a drop in a load-extension response. That change is associated with permanent damage to each mask and friction contact within the rearrangement of loose fibre weaves. Masks with the greatest number of layers reduced particle transmissibility. However, woven or ordered mask fabrics in two layers with different orientations provided comparable performance. Simulation of each mechanical response, velocity streamlining and fibre distribution within the mask layers are also presented.

## Introduction

The current pandemic has led to extensive implementation of mandates to wear face masks. The coronavirus disease 19 (COVID-19) is a respiratory infectious disease caused by transmission of the severe acute respiratory syndrome coronavirus 2 (SARS-CoV-2). SARS-CoV-2 spreads among humans through direct (person to person contact) or indirect contact (i.e., contact with a contaminated surface) with infected individuals or through airborne saliva droplets or nasal secretions generated when an infected person coughs or sneezes^1^. As of May 2022, there had been nearly 518 million confirmed cases and 6 million deaths worldwide^2^. This massive outbreak has led all countries to take measures to contain and mitigate the transmission of the virus. Leading scientists and epidemiologists have predicted that even with the vaccine, the need for masks, hand washing, and social distancing will not disappear. However, during this time, doctors and scientists were able to gather a great deal of evidence about the virus, and now we have more information on how to prevent and treat it more effectively. Experts call for continued use of personal protective and social measures with COVID-19 vaccination. Talic et al. (2021) studies indicated a statistically significant 53% reduction in the incidence of COVID-19 with mask wearing and a 25% reduction with physical distancing^3,4^.

In addition to further measures such as limiting public gatherings, disinfecting surfaces, and the use of hand sanitizer, the world health organisation (WHO) has introduced the use of personal protective equipment (PPE), including face masks^5^. This helps to reduce infections by providing filtration in the blocking of the transmission of the droplets and aerosols generated by infected individuals^6,7^. The increasing demand for face masks has also led to a shortage of commercial supplies. Therefore, to address this shortage, people started to make homemade cloth masks using available fabrics. However, the filtration efficacy of a face mask, and therefore the level of protection against pathogens, depends on different factors such as the size of the airborne particles and their velocity, the fabric material microstructure and the facepiece leakage. These measures show how well a mask prevents the leakage of respiratory air through the facepiece^8,9^. The size of SARS-CoV-2 is in the range of 60 to 140 nm. Thus, the design of the face mask pore size needs to be below this range in order to prevent the passage of the virus^10,11^.

Also, the selection of materials during the design phase of face masks plays an important role in reducing the risk of infection. Cotton has been widely used as its fibres are tightly woven and are able to provide a good level of protection to the wearer due to their low porosity^12,13^. In addition, cotton fibres are made of cellulose, which is a non-synthetic material, so it does not contribute to particle deposition by electrostatic attraction^13^. However, cellulose is a hydrophilic material, and therefore, it absorbs liquids. This feature makes cotton a poor choice as a material for face coverings because, due to its wettability, it can collect and trap viral particles over time on its surface and could be harmful to the wearer^14^. In contrast, polymers such as polypropylene (PP), polyethylene (PE), and polyesters (PL), are synthetic hydrophobic materials that have been widely used for face masks. For instance, PP and PL fibres are used for medical face masks due to their low wettability and non-absorbent properties^15,16^. However, since polymers are synthetic materials, they are not completely breathable fabrics, which can lead to the formation of steam during the respiration cycle, with the mask becoming a reservoir for viral particles. Moreover, synthetic materials can be an irritant to people with sensitive skin^17^. Therefore, the design of face masks consists of selecting and combining layers of different types of materials (e.g., as in surgical masks and N95 respirators)^18,19^.

The surgical face mask is made of three layers, each with a specific function. The inner layer, made of an absorbent material such as cotton, is for absorbing and trapping moisture. The middle layer acts as a filter by stopping the viral particles from penetrating when made of a non-woven and non-absorbent material such as PP. The outer layer, usually made of polyester, is water resistant to repel droplets of fluid. However, Milton et al.^20^ showed that a surgical face mask cannot prevent penetration of particles that are smaller than 5 μm^20^. Du et al.^21^ showed that the N95 respirators (non-woven PP fabrics) are not resistant to oil-based particles. The outer and inner layers are both hydrophobic to prevent the absorption of fluids. The middle layer acts as a filter to capture about 95% of 0.3 μm airborne viral particles^21^. Conflictingly, as filtration efficiency increases, permeability and breathability decrease, i.e., reduced porosity leads to a decrease in air flow through the mask. Furthermore, another study indicated that N95-certified respirators may not provide proper protection against viruses smaller than the 300 nm particle size^22^. In addition, as the inhalation flow rates increase, the efficacy of the N95 respirators can drop below 95%^22^. Clearly, the properties and microstructures that characterise the fabric (i.e., fibre diameter and pore size) can affect the filtration efficiency. Therefore, the aim of this study is to appraise six common certified face masks that have been employed during the pandemic. These include the national health service, England (NHS) approved and certified surgical medical grade IIR, FFP2, FFP3, reusable cotton, antiviral and silk face masks. The appraisal involves microstructural examination (electron microscopy), pore size and fibre diameter measurement, EDX elemental analysis, mechanical strength testing, and cyclic loading for durability assessment. These combined studies and corresponding simulations reveal that multi-layered face masks (FFP2 and FFP3) provide the most effective barrier despite having inferior mechanical properties within individual layers. In contrast, silk, reusable cotton, and antiviral masks offer good strength, protection and durability over a longer period of use.

## Experimental procedure

### Materials

Six commercially available face masks were obtained from NHS-approved suppliers:

- the medical grade IIR ***surgical*** face mask is a lightweight 3-layer with a nose clip. The top and bottom layers are manufactured from spun-bonded polypropylene non-woven fabric. The centre layer is a polypropylene melt-brown non-woven fabric. It is used in the NHS and is EU (European Union) certified.
- the ***reusable cotton*** face mask is washable, reusable and cost-effective compared with other face masks. It is a cloth-based (cotton) mask that has an inserted filter (PM 2.5) for additional protection from ultrafine airborne particles. It is not a certified medical mask and has no mask classifications.
- the filtering facepiece 2 ***(FFP2)*** is an N95 equivalent. It is BSI (British Standards Institution) certified and meets the guidance from the WHO for use during outbreaks or viruses.
- the filtering facepiece 3 ***(FFP3)*** is a certified N99 face mask. It is also a BSI-certified mask that meets the guidance from the WHO for use during outbreaks of SARS, avian flu and coronavirus. It is used in the NHS as well.
- the ***antiviral*** is a reusable face mask that is 100% polyester, anti-viral and anti-bacterial. The inner and outer layers of the fabric are engineered with HeiQ Viroblock, which is an innovative Swiss technology that makes fabrics resistant to harmful microbes.
- the pure mulberry ***silk*** mask is made of 100% Grade 6A (the highest and finest quality silk), is breathable, and is an OEKO-TEX certified product, indicating that the silk is tested for harmful substances to protect health.

## Methods

### Electron microscopy analysis

Scanning electron microscopy (SEM) was carried out using a JOEL instrument (JSM IT200, Japan), operated at an accelerating voltage of 10 kV to study, measure and analyse the size and surface morphology of each face mask structure with high resolution. For the detailed morphological and to identify the elemental composition of materials within the mask structures, energy dispersive X-ray analysis (EDX) was carried out at 10 kV, with the microscope in low vacuum mode and an operating pressure of 50 Pa.

### Strength test (stretch to failure)

Strength tests were performed using an Instron (UK) 30 kN universal tensile testing machine. The wedge jaw clamped 25 mm end lengths of each sample, exposing a test length (L_0_) of between 60–70 mm. The corresponding displacement from jaw separation is recorded simultaneously and plotted against the load as the test proceeds. The machine displays a load (N) versus extension (mm) graph from which stretch rations (λ) are calculated; λ = (L_0_ + x)/L_0_, where x is the extension. This particular test was carried out to examine manual handling during normal day-to-day use, in which the mask is stretched repeatedly as personal protection for the public and professionals. The load is recorded from an Instron calibrated load cell displacement.

### Modelling of hysteresis under cyclic loading

#### (a) Area under the curve

GraphPad Prism version 9.3.1 (GraphPad Prism software, La Jolla, California, USA) was used to evaluate the area under the curves for the medical grade IIR surgical and FFP2 face masks during loading–unloading cycles. The trapezoidal rule was used as a spot check upon the software results. In general, there was good agreement between this rule and the simulation results in identifying those areas required for thermodynamic work/energy analyses.

#### (b) Fibres distribution analysis

SEM images of the six different face masks were used to characterise the morphology of the fibres and structures. In order to analyse the distribution of the fibres across the different layers, 3D fibrous models were generated using Blender 2.9 software (Stichting Blender Foundation, Amsterdam). The 3D models were constructed using the fibre and pore size diameters obtained from the SEM experimental data. Different colours are used to distinguish the multiple layers of each face mask. Simulating the fibre distribution clarified the SEM images and the arrangement of the layered structure within each design, thereby assisting with the global objective of quantifying filtration efficiency.

## Results and discussion

### Microstructural evaluation

**Figure 1:**
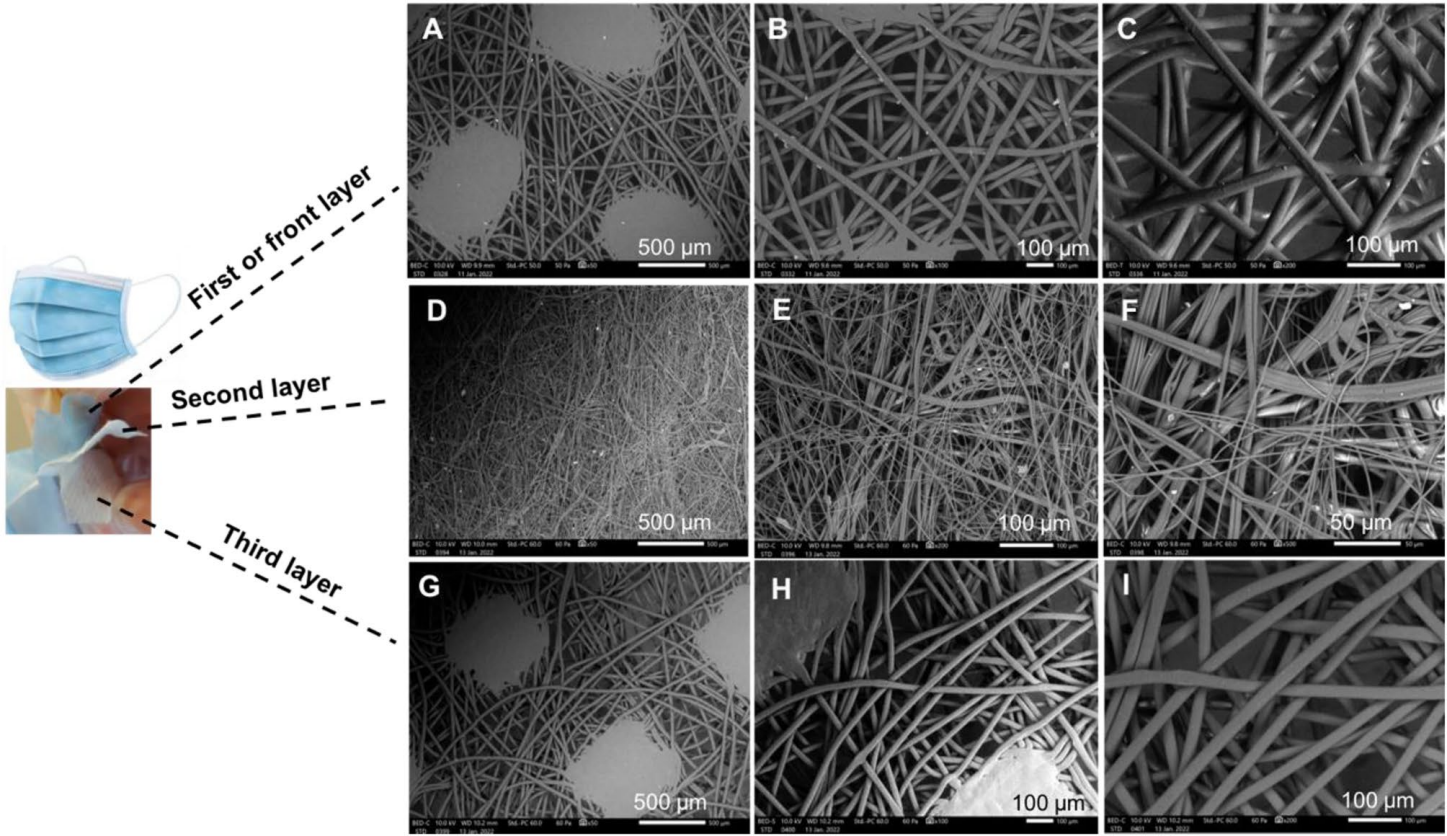
SEM for each of the three layers of a surgical mask. (**A**–**C**) front layer. (**D**–**F**) second layer. (**G**–**I**) third layer.

The effectiveness of face masks depends on their fibre structure, morphology, and porosity. Fabrics of high filtering efficiency and low airflow resistance would perform effectively in blocking airborne particles^16,19^. They serve to provide the best air permeability as well as enable the mask users to breath freely. The disposable certified medical grade type IIR (2R) ***surgical*** face mask (surgical) (Fig. 1) consists of three layers shown at increasing magnifications (50, 100, and 200 µm). The regular hot spot welding appears at a 50 µm magnification. The PP fibre details appear at the higher magnifications (100 and 200 µm). The three layers have a random orientation; the top and bottom layers are manufactured from spun-bonded polypropylene non-woven fabric, and the centre layer is melt-brown polypropylene non-woven fabric.

When overlaid, the three layers maintain an overall pore size of 1570 µm^2^. However, if the fibre orientation was less random, the effective pore size is equalised and can be expected to alter the barrier resistance. Thereby, in a finer weave, the passage of viruses or particles from sprays or splashes (i.e., from sneezes and coughs) is better resisted. The requirement here is that the particle size be greater than the fibre pore size.

In addition, transmissibility efficiency (%) was estimated at 95% from the airflow velocity measurement through the three layers using Eq. 1, where v_0_ is at entry, v_1_ is at the exit of the mask layer^23^.

1 $$Transmissibility \, efficiency = \frac{vo - v1}{{vo}}$$

Other measures of airborne transmission for masks in normal use when fitted properly to provide optimum protection efficiency have been studied by other scientists. Other researchers have used i) aerosol concentration as an alternative measure of particle penetration; ii) the application of Darcy’s law using the flow rate; and iii) leakage analyses^24–26^.

An estimate of the total area of the pores within the SEM probe area for the first layer is seen to be 21% (i.e., within an area of 0.3 mm^2^). This amount of porosity is assumed as an average for the whole layer given the uniformity of the texture’s structure.

**Figure 2:**
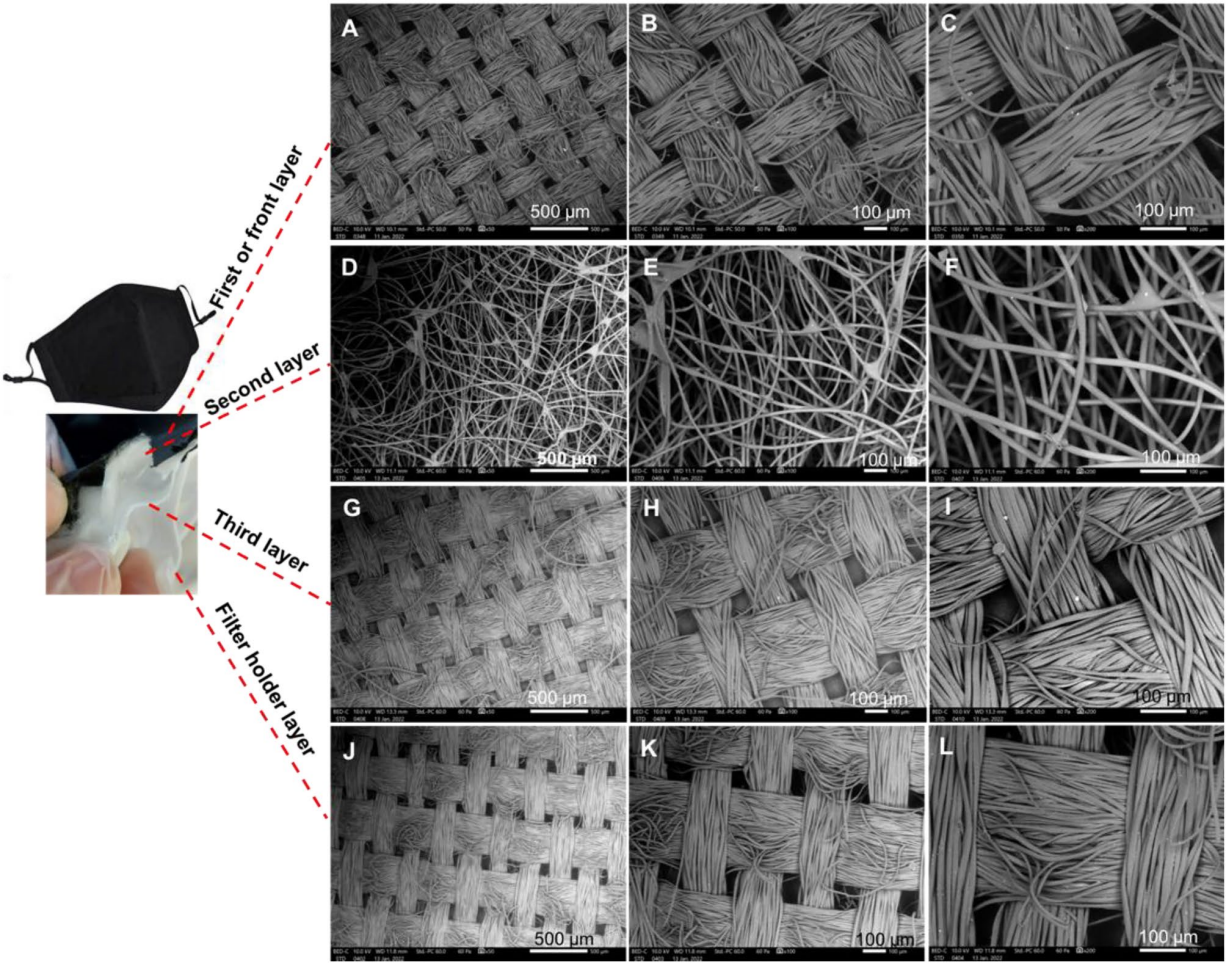
SEM for each of the four layers of a reusable cotton mask. (**A**–**C**) front layer. (**D**–**F**) second layer. (**G**–**I**) third layer. (**J**–**L**) Filter holder layer or back layer.

Figure 2 shows the scanning electron microscopy at 50, 100, and 200 μm magnifications of a woven fibre, reusable cotton mask which contains three layers and a filter holder.

The entire mask is made of 70% cotton and 30% spunlace, as recommended by the WHO. Various filters (i.e., PM 2.5) can be added to the mask filter holder, offering further protection from fine particles in the air. An ordered orientation of the first, third, and filter holder layers can be seen in Fig. 2A–C, and G–L.

The first three layers are held together by a fold, and the filter holder is carried by the last layer. Using Eq. 1, the preliminary filtering efficiency of this mask was measured as 96%^23^. The total area of the pores within the SEM probe area for the first layer is estimated at 24% (i.e., within an area of 0.3 mm^2^, the porosity was 24%).

**Figure 3:**
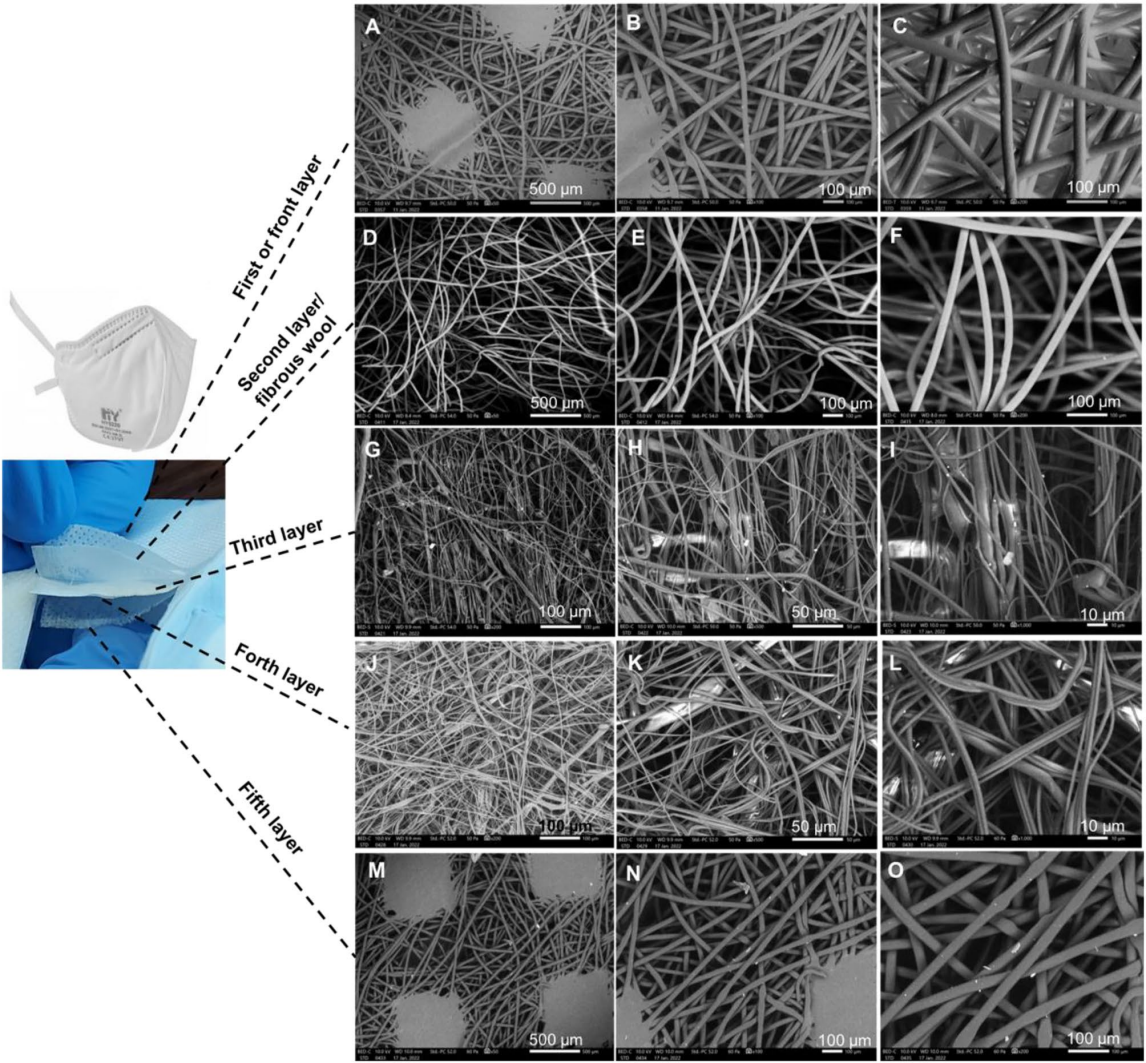
SEM for each of the five layers of a FFP2 mask. (**A**–**C**) front layer. (**D**–**F**) second fibrous layer. (**G**–**I**) third layer. (**J**–**L**) forth layer. (**M**–**O**) fifth layer.

The ***FFP2*** mask (Fig. 3) is an N95 equivalent and certified disposable face mask which meets the guidance from the WHO and the European ***FFP2*** standard for use during outbreaks or viruses (i.e., SARS, Avian Flu). It is a flat-folded style respirator mask with inner and outer layers that are manufactured from high-quality polypropylene materials with a filter material of fibrous wool, providing high protection and low breathing resistance during use (Fig. 3).

This mask consists of five layers shown at increasing magnifications (50, 100, and 200 µm). The hot spot welding appears at the 50 µm magnification and the fibre details appear at the higher magnifications (100 and 200 µm).

The random orientation of the layers arises from spun-bonded polypropylene manufacture, giving a non-woven fabric with an average porosity of 1603 µm^2^ and an average fibre diameter of 14.4 µm.

The preliminary transmissibility efficiency of this mask was calculated at 98% using Eq. 1^23^. An estimate of the total area of the pores within the front layer was found to be 16% based upon the SEM prob area.

**Figure 4:**
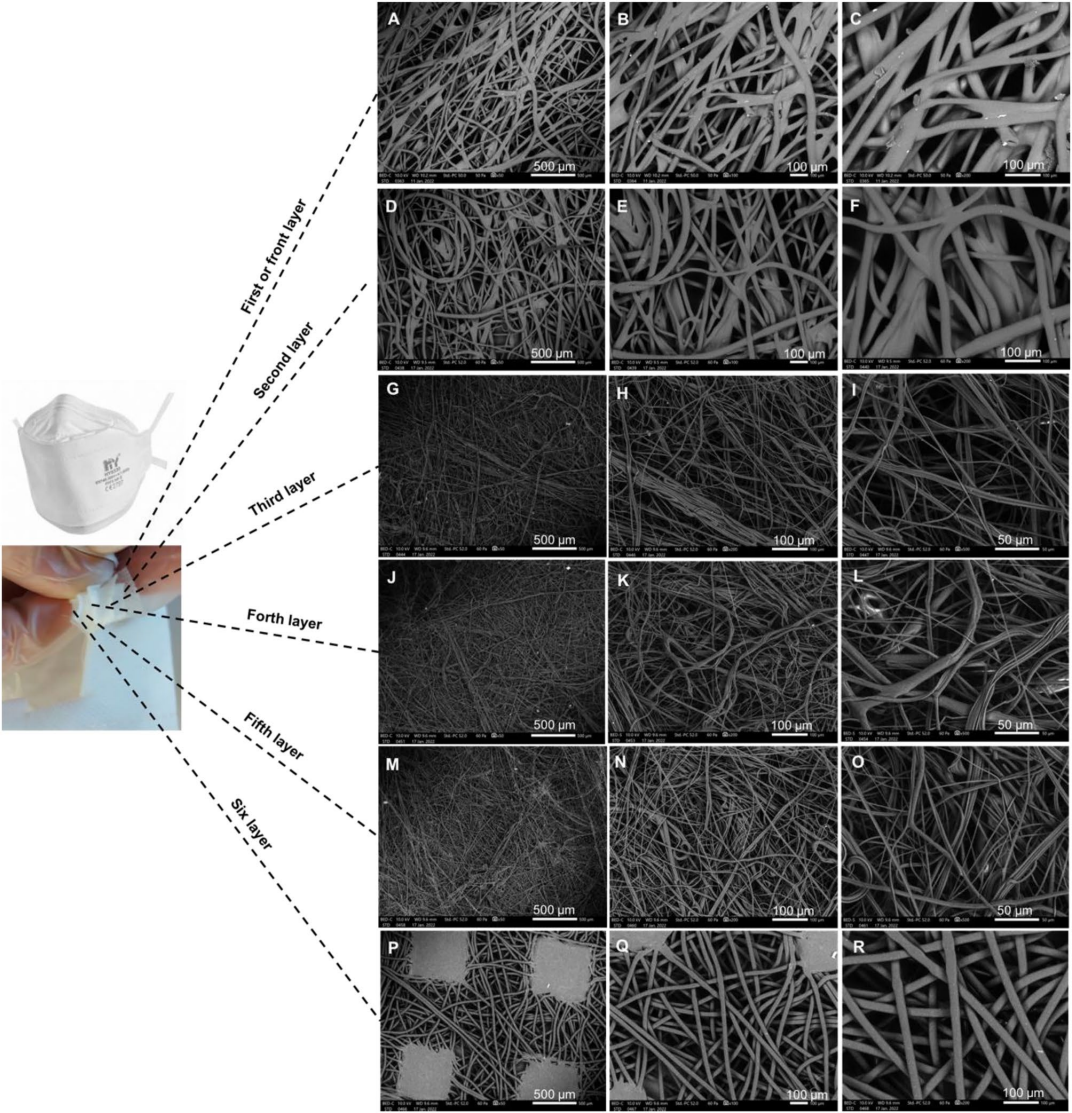
SEM for each of the six layers of a FFP3 face mask. (**A**–**C**) front layer. (**D**–**F**) second layer. (**G**–**I**) third layer. (**J**–**L**) forth layer. (**M**–**O**) fifth layer. (**P**–**R**) six layer.

The ***FFP3*** mask (see Fig. 4) is a folded flat design, consisting of a disposable respirator and a certified (N99) face mask. The mask is BSI and EU certified as a standard grade face mask. This mask meets the guidance given by WHO for use during the outbreaks (i.e., SARS, Avian Flu, and COVID). It has also been used by the NHS as a reliable and effective protection during the pandemic. It has a 3D structure and a large filtration area with polypropylene inner and outer layers and a non-woven fabric that provides a smooth lining.

This mask consists of six layers shown at increasing magnifications (50, 100, 200, and 500 µm). The hot spot welding appears at a 50 µm magnification and the fibre details appear at the higher magnifications (100–500 µm).

The overall filtering efficiency of this mask was also calculated at 98% using Eq. 1^23^. An estimate of the total area of the pores within the front layer was found to be 11% based upon the SEM prob area.

**Figure 5:**
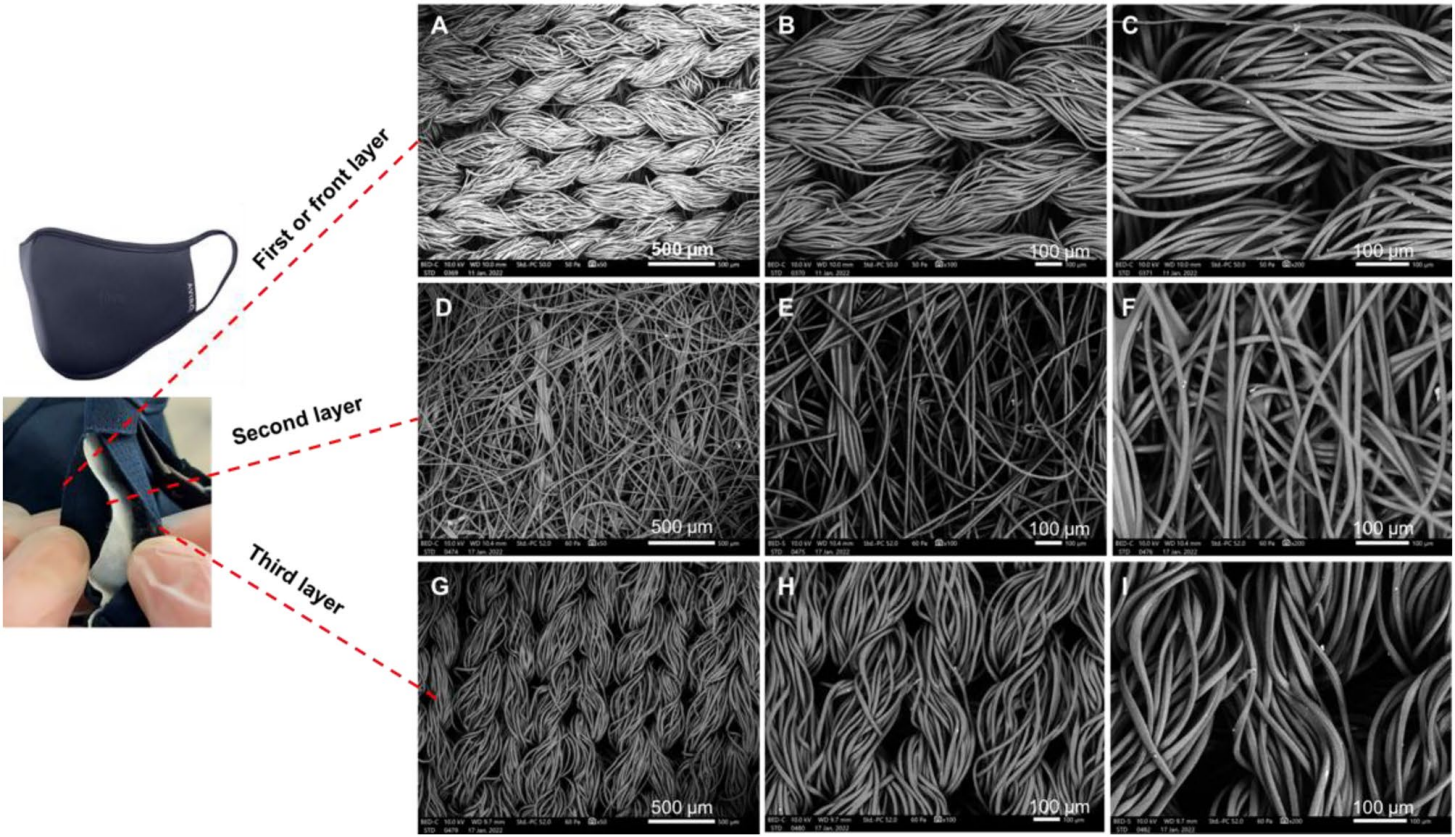
SEM for each of the three layers of an antiviral face mask. (**A**–**C**) first or front layer. (**D**–**F**) second layer. (**G**–**I**) third layer.

The three-layered ***antiviral*** face mask (see Fig. 5) is made of 100% reusable polyester and is both anti-viral and anti-bacterial. These provide a washable face mask. Face masks can be a dangerous reservoir of viruses and bacteria when touched during application and removal, risking the transfer of pathogens.

This face mask is an anti-viral (coronavirus & H1N1) and its anti-bacterial efficiency is > 99% based on ISO 18,184 (MSL Labs UK) and ISO 20,743 (Microbe Investigations AG Switzerland). This is due to the self-sanitizing nature of the anti-bacterial treatment on the fabric using an active ingredient of silver chloride. The randomly orientated middle layer lies between two outer layers (inside and outside) and assumes the usual rules of a filter. Due to its self-sanitizing property, this face mask does not need to be washed regularly as it is not intended for medical use. This product is also treated with a biocide to protect it from spoilage by microbes and germs.

This mask consists of three layers shown in Fig. 5 at increasing magnifications (50, 100, and 200 µm). The first and third layers are of woven ordered structure. An estimate of the total area of the pores within the front layer was found to be 3% based upon the SEM prob area.

**Figure 6:**
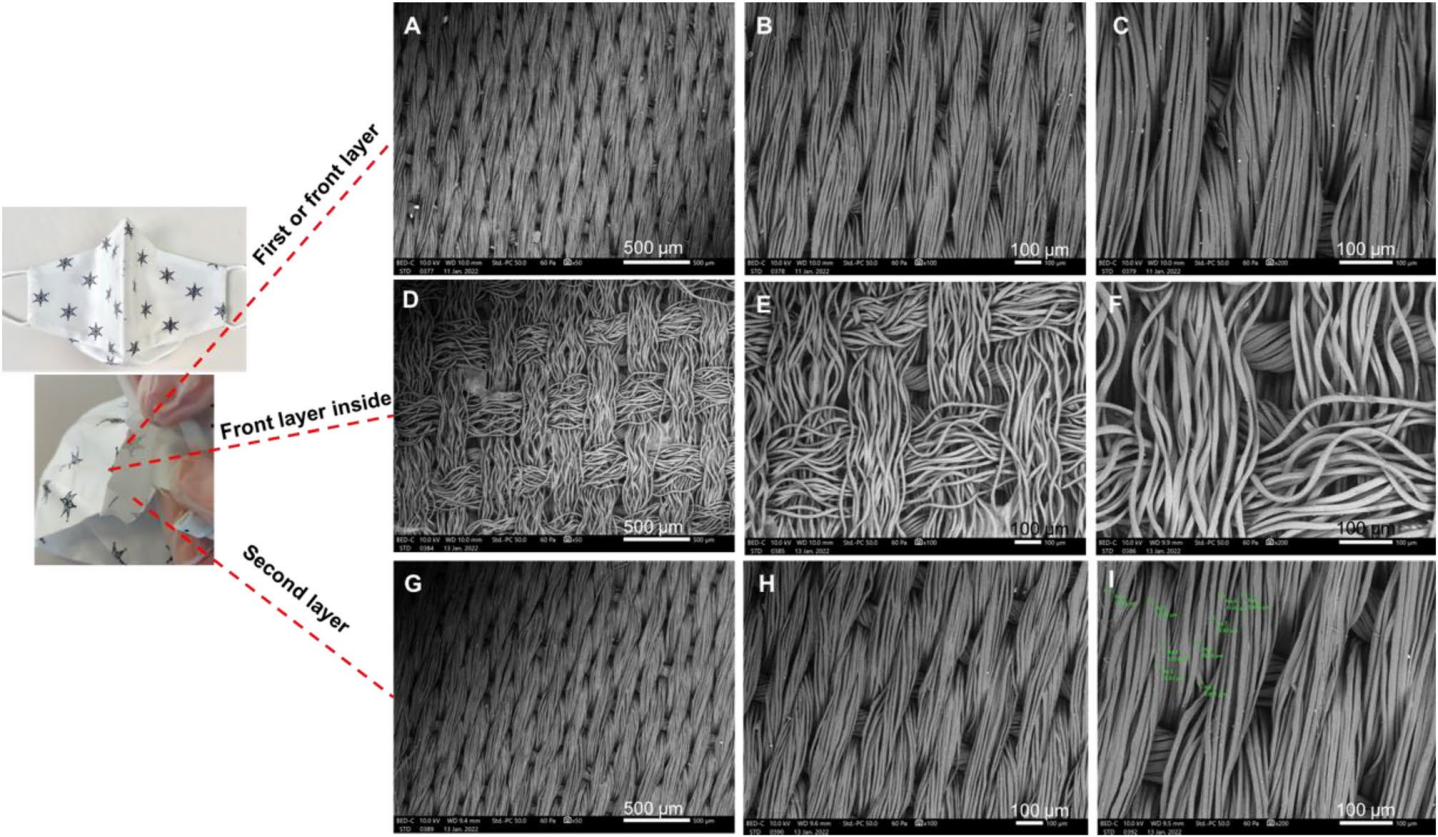
SEM for each of the two layers of a silk face mask with nose wire**-** pure mulberry silk breathable. (**A**–**C**) front layer. (**D**–**F**) the back of front layer. (**G**–**I**) second layer.

Figure 6 shows the 100% breathable mulberry ***silk*** face mask. The ***Silk*** face mask is handmade using pure organic mulberry silk. They incorporate a nose wire and adjustable, soft-elasticated﻿ earrings. They are also washable and reusable. The fold within the material weave is arranged to orientate the overlaid fibres more randomly between its two layers, thereby improving barrier resistance.

This mask consists of two layers shown in Fig. 6 at increasing magnifications (50, 100, and 200 µm). Figures 6 A–C and G–I show the front layer’s two sides. Both sides appeared ordered, layered in a woven spiral structure (Fig. 6A–C front view (outside); Fig. 6D–F back view (inside). The front layer’s inside shows some irregularity within a looser crossed weave.

Air flow velocities at entry and exit showed that the filtering efficiency of this mask was calculated at 96% (from Eq. 1)^23^. An estimate of the total area of the pores within the SEM probe area of the front layer was found to be 2.5%, which in a regular weave may be assumed across the whole area of this layer.

### EDX elemental analysis

**Figure 7:**
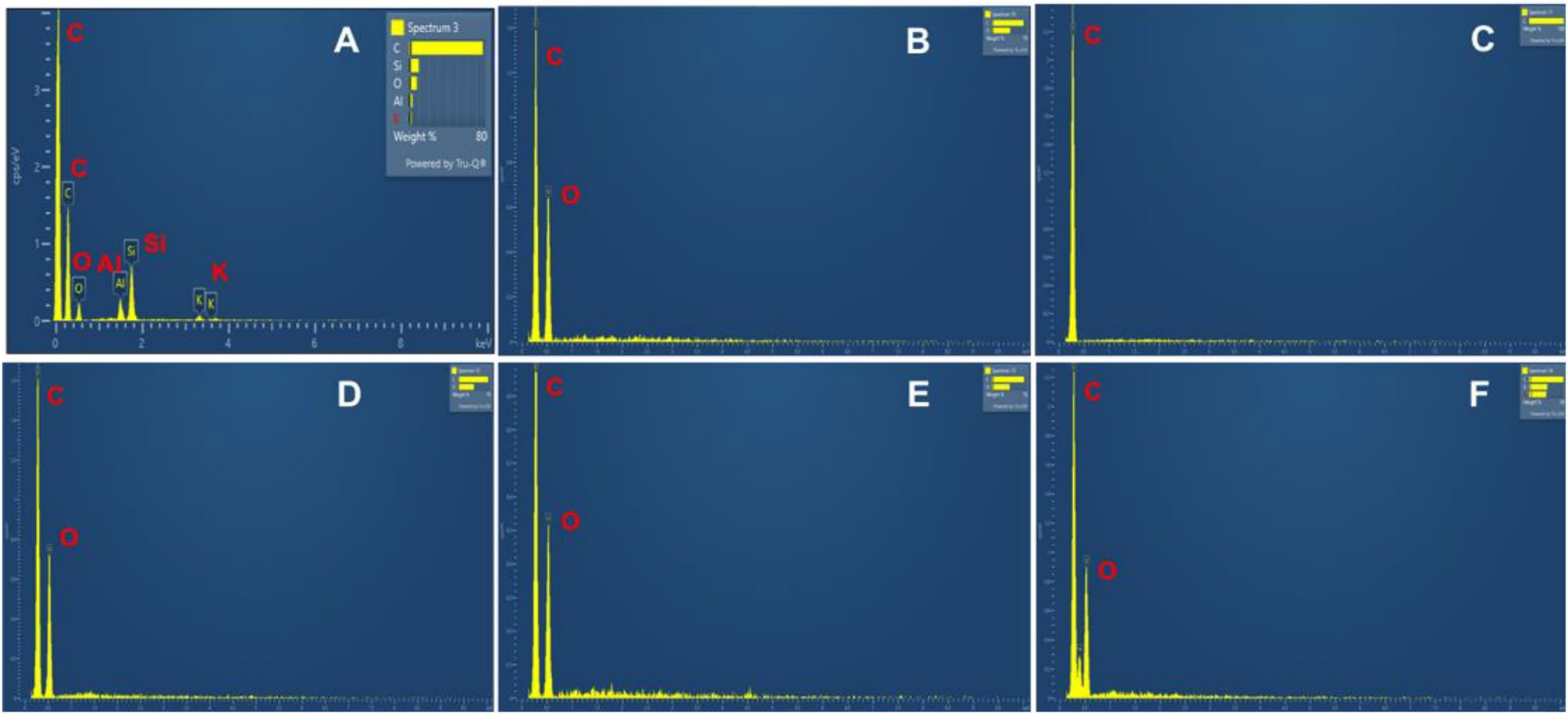
EDX elemental analysis of the six approved face masks. (**A**) surgical. (**B**) reusable cotton. (**C**) FFP2. (**D**) FFP3. (**E**) antiviral. (**F**) silk.

The entanglement of fibres for the ***surgical*** mask, having an average diameter of 16.3 µm, defines each fabric thickness of less than 0.5 mm. The hydrocarbon molecular chain is revealed by the energy dispersive X-ray analysis (EDX) adapted for elemental analyses of the masks. Additional probing revealed the following impurities at different positions: calcium, oxygen, aluminium, silicon, potassium, and carbon in the quantities shown in Fig. 7A. These spectra reveal the typical spread of impurities within each probe area investigated (0.3 mm^2^). The three layers with folds are held in place centrally by the hot spot welding and by glueing around the boundaries, processes which can introduce the resulting impurities within the layers. Figure 7B shows carbon, along with hydrogen, as the dominant elements for the ***reusable cotton*** mask. The ***FFP2*** mask demonstrated carbon (along with hydrogen) as the dominant element, as was expected for a hydrocarbon chain (see Fig. 7C). Also, Fig. 7D for the ***FFP3*** mask revealed oxygen with carbon (along with hydrogen) as the dominant elements. The ***antiviral*** face mask analysis (see Fig. 7E) confirmed oxygen, carbon, and hydrogen were present in the chemical composition of the fibre.

The ***silk*** mask analysis (Fig. 7F) showed oxygen and nitrogen, along with carbon and hydrogen, as the constituent elements. The EDX analyses show carbon as a dominant element in all six masks. Atmospheric oxygen plays a secondary role in all probes. The trace elements; aluminium, silicon and potassium are also appeared within the surgical mask.

### Fibre and porosity analyses

The ***surgical*** mask appears with an approximately uniform average porosity of 1570 µm^2^ and an average fibre size of 16.3 µm. The structures remain random for the second and third layers, with different fibre diameters of 3.5 µm and 21.6 µm, and pore size of 181 µm^2^ and 2207 µm^2^ correspondingly. The ***reusable cotton*** mask’s layers have a near uniform average pore size of 1547.5 µm^2^ and an average fibre diameter of 16.3 µm. The structure for layers (one, two and filter holder) remains ordered with different fibre diameters of 23.85, 3.46, and 21.60 µm and pore sizes of 2254.5, 180.7, and 2207.3 µm^2^ respectively. The second layer is made of wool with a fibre dimeter of 12.5 µm and a pore size of 1057 µm^2^.

The ***FFP2*** mask’s porosity and fibre diameter of the third and fourth layers are 63 µm^2^, 1.8 µm and 39 µm^2^, 2.2 µm respectively. The reduced porosity appears with the greater density of these overlaying layers. The ***FFP3*** mask’s random orientation of all six layers arises from spun-bonded polypropylene manufacture similar to the ***FFP2*** mask with an average porosity of 912 µm^2^ with an average fibre diameter of 14 µm. The porosity and fibre diameter of the third, fourth, and fifth layers are: 265 µm^2^, 2.1 µm; 46 µm^2^, 2.5 µm and 89 µm^2^, 2.5 µm respectively.

The ***antiviral*** mask’s random orientation of the second layer at similar magnifications appears in greater density with an average porosity of 3484 µm^2^ with an average fibre diameter of 12 µm. The porosity of the second layer is 608 µm^2^ with a fibre diameter of 11.7 µm. The decreased porosity and random orientation of smaller diameter dense fibres serve to increase this layer’s barrier resistance. The ***silk*** mask’s pore size is consistent between its two layers (3013 µm^2^). Fibre diameters of 10.6 and 11.7 µm, as measured, apply to the first and second layers, respectively, indicating that the two ***silk*** layers are approximately identical.

**Figure 8:**
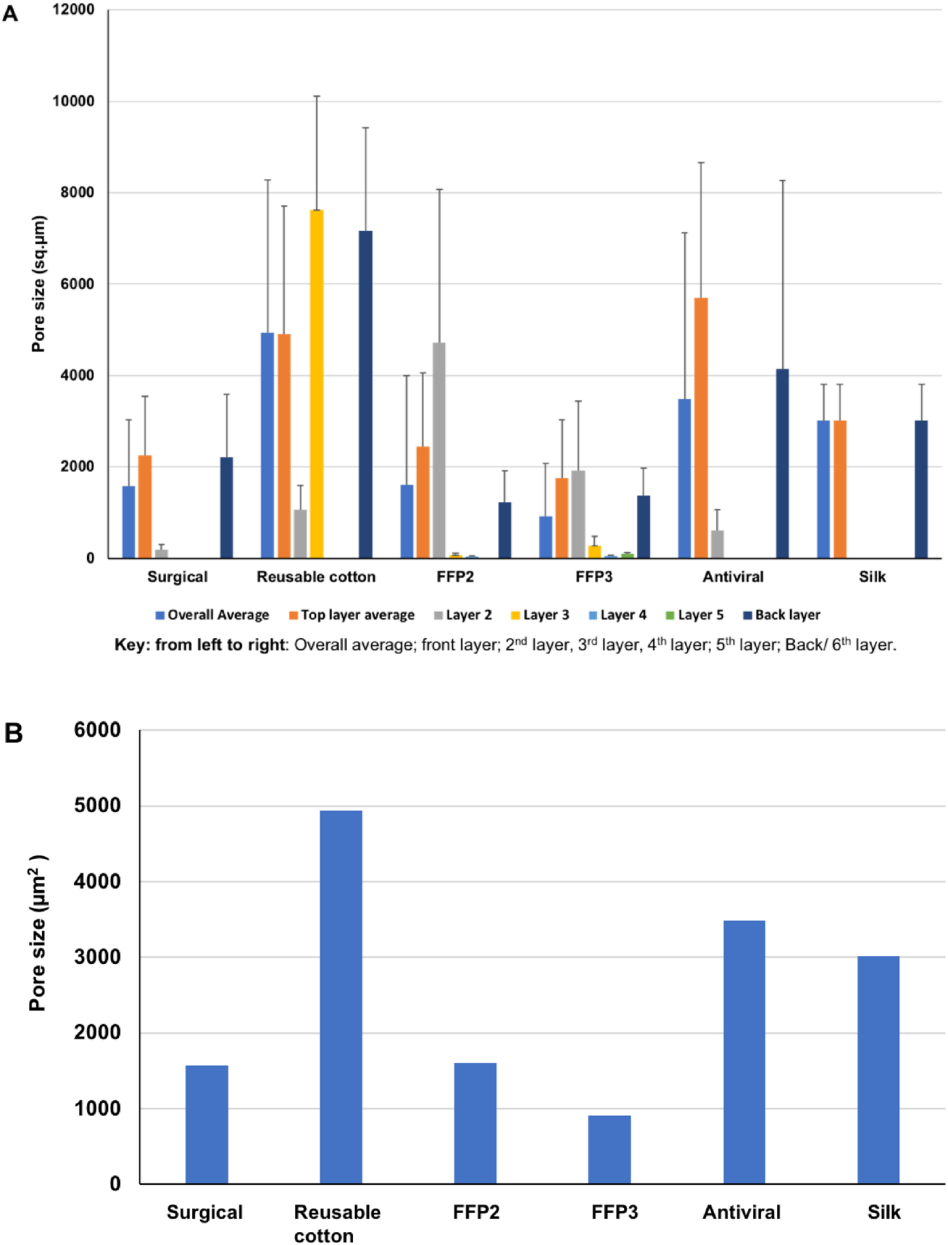
The pore size for the six masks was derived from individual layer measurements. (**A**) for each layer. (**B**) average pore size of each mask.

**Figure 9:**
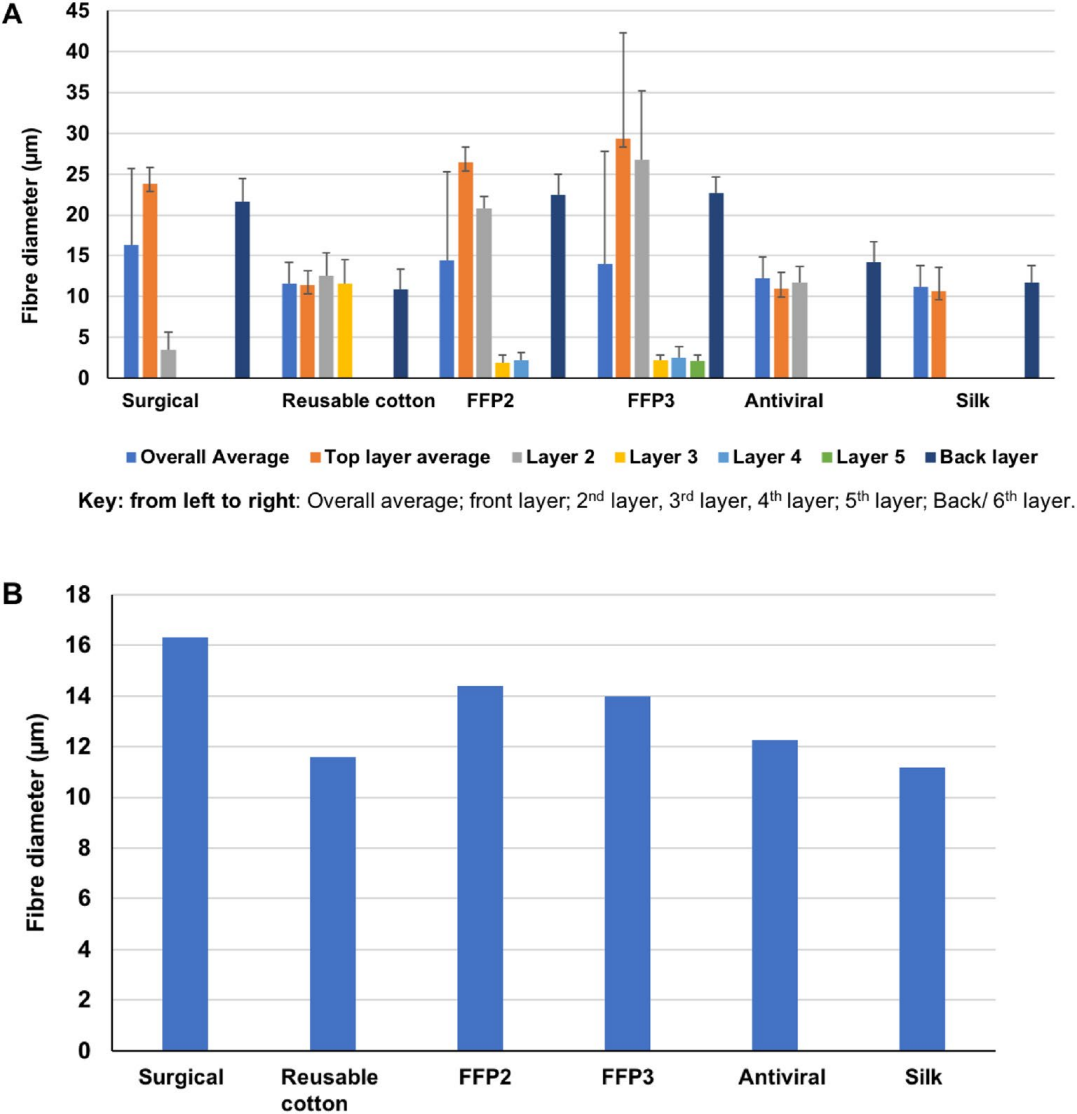
The fibre diameter for the six masks was derived from their individual layer measurements. (**A**) for each layer. (**B**) average fibre diameter of each mask.

It is the crossed orientations of the weave within the folded design that has enhanced this mask’s barrier resistance (see Fig. 8 and 9). The error bars for pore size indicate variation in measurement based upon 10–15 probe counts for individual layers and the whole mask. Here, the number of layers depends upon the mask. Figures 8A and 9A (bar charts) show the pore size and fibre diameter, respectively. This includes the number of layers in each mask, as well as the overall assembly. Figures 8B and 9B show the average porosity and average fibre diameter of each mask as a whole. It is evident from Fig. 8B that mask porosity varies considerably. In contrast, fibre size is relatively constant (Fig. 9B). This mismatch suggests an assessment of mask performance in which the enhanced density of the fabric offers greater filtration efficiency. On this basis, the ***FFP2*** and ***FFP3*** masks, coupled with their greater number of layers, lead the others.

### Mechanical behaviour

Relatively few investigations have been conducted on the mechanical properties of face mask materials. Cotton and PP fibres appear in a few publications with different applications under dynamic loading^27^. The durability of face masks in handling and wear has been given by Varanges et al.^28^. The effect of the mechanical behaviour of each mask was assessed in its strain or stretching behaviour during wearing. For this, the strength test (stretch to failure) and repeated loading tests were conducted on single and multi-layers.

**Figure 10:**
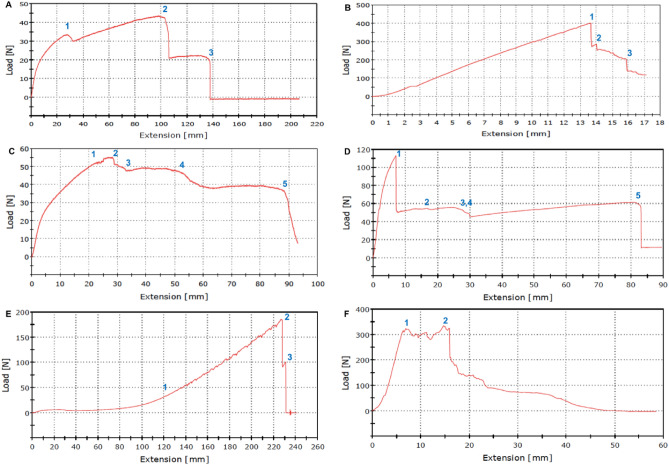
Strength test (stretch to failure) for multi-layered mask materials. Key: (**A**) surgical. (**B**) reusable cotton. (**C**) FFP2. (**D**) FFP3. (**E**) antiviral. (**F**) silk.

Figure 10A (***surgical*** mask) shows the strength test results (at the rate of 25 mm/min) indicated by load versus displacement plots.

The points 1, 2, and 3 indicate the load at which individual fibres fail. In Fig. 10A, the greatest stretch ratio at ~ 3 arises within the most elastic front layer, which is the last to fail. However, the user should not exceed a stretch of 1.4 if the mask is not to be damaged by the wearer.

2 $$\lambda = \frac{Lo + x}{{Lo}} = 1 + \frac{x}{Lo}$$

The events (1–3) represent stretch ratios $(\lambda )$ from Eq. 2 (with respect to the original test length) of 1.4, 2.46, and 3 respectively.

Figure 10B (***reusable cotton*** mask) indicates the load at which each of the three individual fibres fails, where the wool layer was the first to fail. The greatest stretch ratio ($\lambda$) at around 1.25 arises from the cotton front layer with much reduced elasticity. Here, the safe stretch ratio of 1.20 at point (1) applies. The points (1–3) correspondingly to stretch ratios $(\lambda )$ of 1.20, 1.22 and 1.25 respectively. Figure 10C (***FFP2*** mask) demonstrated the points (1–5) at which each of the five individual layers failed. The greatest stretch, at around 95 mm arises from the ***FFP2*** with increased elasticity. Here, however, the safe stretch ratio is limited at point (1) to 1.40, at which the third and fourth layer failed together. The remaining events (2–5) represent stretch ratios $(\lambda )$ of 1.50, 1.60, 1.95, and 2.6 respectively.

Figure 10D (***FFP3*** mask) showed the points (1–5) at which five of the six individual layers failed, leaving the first layer unbroken. The stretch ratio is limited to 2.2 at point (5). At points 3 and 4, the third and fourth layers failed together ($\lambda$=1.43). The stretch sequence (1–5) corresponds to stretch ratios of 1.1, 1.26, 1.43, 1.43, and 2.2 respectively.

The ***antiviral*** mask stretch test shown in Fig. 10E revealed the first failure in the middle layer (second layer) at points 1, $(\lambda$=2.6). Thereafter, the two identical outer layers stretched together to fail at points 2 and 3 (for a stretch ratio of $\lambda$=3.85). The ***silk*** mask stretch test shown in Fig. 10F revealed the ripping of the woven structure in the region 1–2 at the maximum load of 330 N and a limiting stretch ratio range of 1.1–1.3. Also, the stretch ration at failure is approximately 1.9 ($\lambda$). This demonstrates that the strength of the weave compromises its elasticity. The overall accuracy of each test depends entirely upon the instrument measurement methods. Readings from the calibrated load cell and cross head displacement were equated to test piece loading extension. The accuracy was estimated at ± 5 mm and ± 10 N for displacement and force measurement, respectively. These were judged to be sufficiently accurate given the ranges of loading and the large amount of stretch involved in each test.

**Figure 11:**
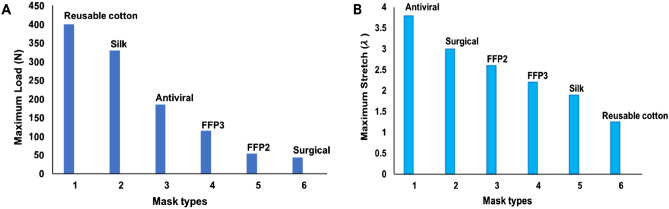
Mechanical properties of approved face masks. (**A**) maximum load. (**B**) stretch to failure.

Figure 11A,B provide a comparison between the strength and stretch for all the masks investigated in this study. They demonstrate that the ***reusable cotton*** mask can withstand the greatest load of 400 N while exhibiting the least stretch (λ = 1.25). The ***silk*** mask offers a comparable performance with increased stretch under reduced load.

In contrast, the ***surgical*** mask offers good stretch at a stretch ratio of three $(\lambda )$ but with little strength. The ***FFP2*** and ***FFP3*** masks offer a mid-range performance in both their strength and stretch assessments.

The ***antiviral*** mask is superior to all mask designs in terms of maximum stretch capacity (twice that of silk), extending by 3.85 times its original length (λ = 3.85). The maximum load capacity of 200 N is inferior to that of a reusable cotton mask (see Fig. 11A,B).

### Modelling of hysteresis under cyclic loading

Zrida et al. (2016) simulated the loading and unloading tests with several cycles, indicating good agreement to exist between the experimental and numerical data obtained in their cyclic tests. Therein, the hysteresis loop energy was relatively well described for all tests for the two materials (PP and alfa/PP)^29^.

**Figure 12:**
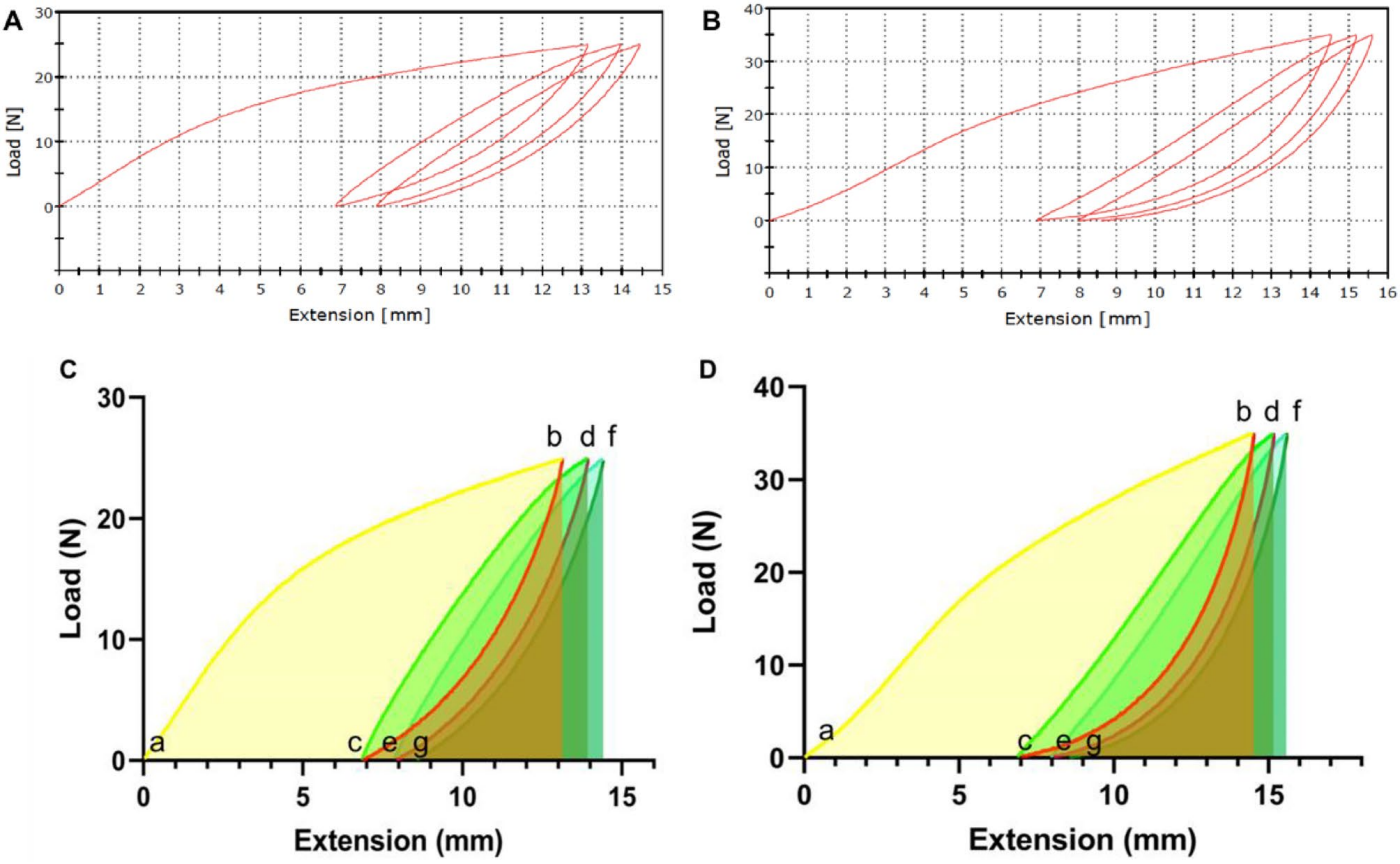
(**A**) Surgical and (**B**) FFP2 experimental hysteresis plots. (**C**–**D**) Simulation of the area under the curve (unloading-loading cycles) for (**C**) surgical and (**D**) FFP2 face masks. [Area Unit: N x mm = mJ (millijoule)].

Figures 12A,B provide our examples of hysteresis loops that apply to cyclic loading of ***surgical*** and ***FFP2*** mask materials measured in the Instron machine described earlier for the strength testing section. When loading and unloading lie between constant loads, it is seen that a shift occurs along the displacement access where loading lags unloading within each cycle. Restricted to the ***surgical*** and ***FFP2*** masks, the areas under the cyclic loading curves (ab, cd, and ef) in Figs. 12C,D simulate the energy absorbed by each mask material when stretched. Contrary to this, the area under the unloading curves (bc, de, and fg) in Figs. 12﻿C,D represents the energy released by the materials (mJ).

The area enclosed within the hysteresis loops (bcd and def) represents the input work absorbed by each mask during each loading–unloading phase (mJ).

This is manifested in the deformation and rearrangement of the random fibre pattern or ordered weave, indicating an irrecoverable permanent stretch. Table 1 shows that those areas beneath the loading and unloading graph are in the ratio of 2 to 1, indicating consistently that one half of the input work is absorbed in mask deformation for each unloading-loading cycle (i.e. work ratios: b–c/c–d ~ ½ and also d–e/e–f ~ ½). The plots show work done, energy released, and energy absorbed (hysteresis) within three loading–unloading cycles to one-half maximum load capacity.

**Table 1:** Enclosed areas under the loading and unloading curves for the surgical and FFP2 face masks. The energy loss has been calculated and simulated for each hysteresis loop for both masks.

Area below: (mJ)	Surgical Mask (Simulation)	Surgical Mask (Calculated)	FFP2 Mask (Simulation)	FFP2 Mask (Calculated)	Energy (mJ)
a–b (1st L)	219.47	218.69	302.94	302.15	Absorbed
b–c (1st UL)	53.54	53.50	70.84	70.85	Released
c–d (2nd L)	105.28	105.14	150.28	150.34	Absorbed
d–e (2nd UL)	52.43	52.36	68.37	68.37	Released
e–f (3rd L)	94.91	94.98	134.25	134.25	Absorbed
F–g (3rd UL)	50.94	50.90	67.13	67.54	Released
**Energy loss (mJ)**					
abc (E)	161.14	165.19	226.25	231.30	1st cycle
cde (E)	52.85	52.78	81.92	81.97	2nd cycle
efg (E)	43.96	44.08	67.12	66.71	3rd cycle

*L* loading; *UL* unloading; *E* enclosed area.

The values of the different areas so identified were obtained using GraphPad software. They are shown in the table below (Table 1). The values of energy loss for each hysteresis loop have been calculated as the difference between the areas under the loading and unloading curves for each of the two face masks. In order to provide evidence of the agreement between the manual and simulation methods, the area under the curve was calculated manually using the trapezoidal rule. The similarity between the data allows us to state that there is excellent agreement between the two methods for providing the areas.

### Fibre simulations

**Figure 13:**
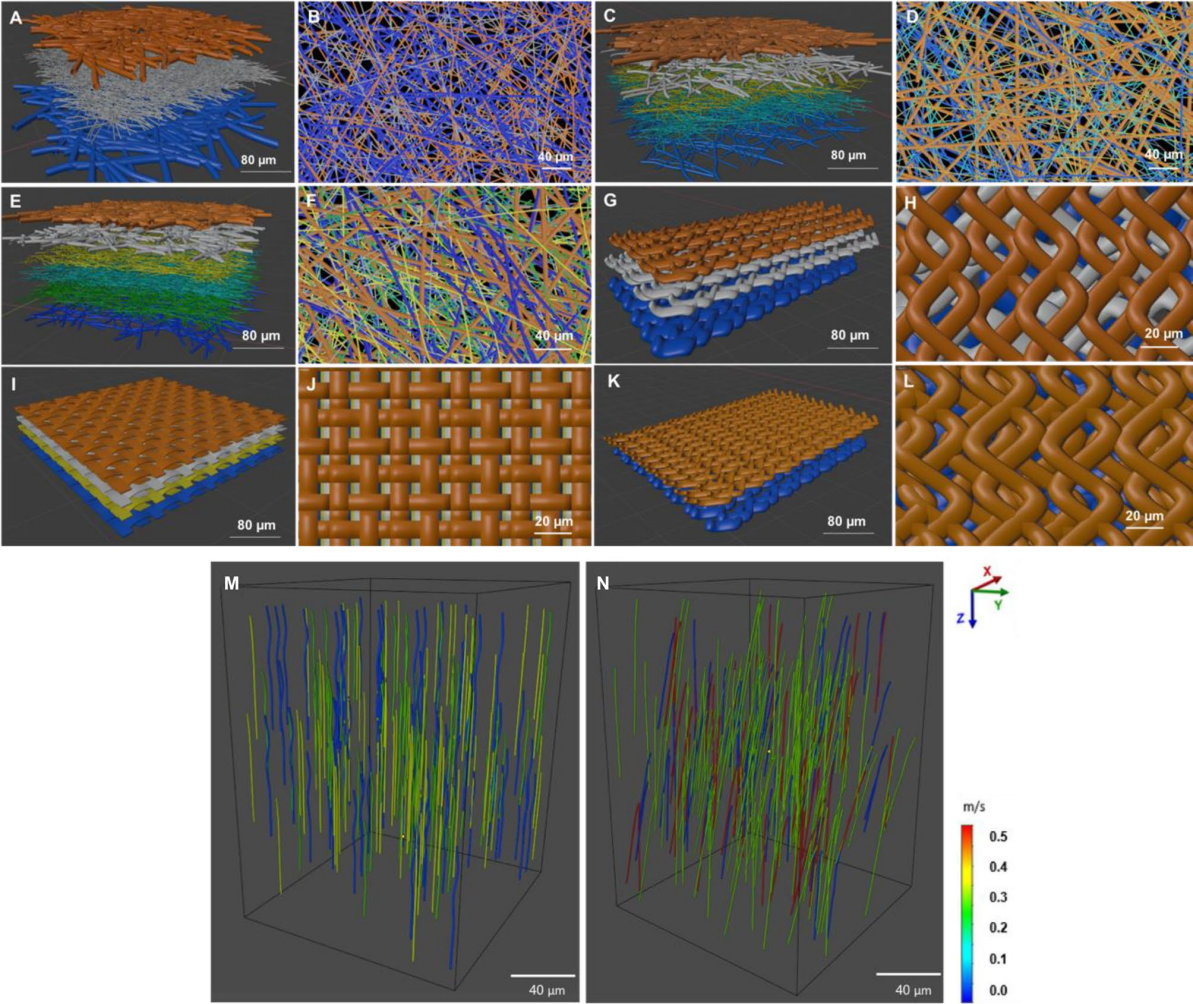
Simulation of fibre distribution of all layers for face masks: (**A**, **B**) surgical. (**C**, **D**) FFP2. (**E**, **F**) FFP3. (**G**, **H**) reusable cotton. (**I**, **J**) antiviral. (**K**, **L**) silk. The velocity streamline appears in the z-direction for the layers of two face masks: (M) surgical and (N) FFP2.

Lee et al. (2021) simulation studies indicated the effects of the filter microstructure and ambient air condition on the aerodynamic dispersion of sneezing droplets. They demonstrated with a micro-to-macroscale bridging approach that wearing a face mask could reduce the transmittance distance of droplets depending on the mask type^30^. Figure 13A–L shows the layers of each face mask along with the fibre distribution in each layer using Blender 2.9 software. The ***surgical***, ***FFP2***, and ***FPP3*** face masks demonstrate a random distribution of the fibres across the different layers (Fig. 13A–F). Conversely, the ***reusable cotton***, ***antiviral***, and ***silk*** face masks exhibit a woven and ordered texture (Fig. 13G–L).

The overall structure of the ***surgical*** face mask (Fig. 13A,B) shows large gaps between the fibres despite the high fibre density in the second layer (grey). In contrast, the ***FFP2*** (Fig. 13C,D) and ***FFP3*** (Fig. 13E,F) face masks show higher fibre density and lower pore size, especially towards the back layers. The third and fourth layers of the ***FFP2*** face mask (yellow and light blue, respectively) demonstrate a compact fibre composition, while in the ***FFP3*** face mask, the third, fourth, and fifth layers (yellow, light blue, and green, respectively) show higher fibre density, thus creating a compact matrix of fibres with a low pore size.

The woven ***silk*** face mask (Fig. 13K,L) shows an overall low pore size, a small fibre diameter, and a consistent fibre density between its two layers. Also, the reusable cotton (Fig. 13G,H) exhibits a low fibre diameter but with larger gaps between fibres.

The interest in simulating the fibre distribution is to clarify the SEM images and the arrangement of the layer structure within each mask. For instance, the simulations make it easier to distinguish the different layers and the geometrical structure of the fibres, whereas the images obtained through the SEM microscope are greyscale images. Thus, the simulation of the fibre’s structure and distribution is to show how each mask layer lies within the whole mask. Figure 13 shows two different orientations for each mask: a trimetric view (Fig. 13A,C,E,G,I,K) and a top view (Fig. 13B,D,F,H,J,L). Two different orientations were chosen in order to emphasise the different geometrical properties between the face mask layers. The trimetric views show the geometrical properties of each mask layer. In particular, these views exhibit the fibre density distribution in the layers. The top views show how the fibres sit upon each other, revealing the porosity and fibre diameter.

Furthermore, using Blender 2.9 software, the fibre simulations have been used to evaluate the velocity streamline across the layers of the ***surgical*** (Fig. 13M) and ***FFP2*** masks (Fig. 13N). The inlet velocity was set to 0.1 m/s to simulate the exhalation velocity for nasal breathing^30,31^ and spherical particles with a size of 5 µm have been considered for this analysis based on previous studies^32,33^. The velocity travels along the z-axis, from the top layer to the bottom layer for each face mask. The Navier–Stokes equation was used to predict the motion of the air flow across the face mask layer^34^. The simulation results show a higher density velocity field at the middle layers of the ***FFP2*** compared to the surgical. This difference occurs because the middle layers of the ***FFP2*** (Fig. 13C, yellow and light blue) are made of highly compacted fibres with low pore size and small diameters, thus creating a dense thick layer. The simulations have been performed as a preliminary study on the impact of two face mask structures on the velocity profile. Further filtration efficiency studies are continuing especially as the COVID virus remains a threat to the world population.

## Conclusion

This study involving microstructural and mechanical properties of approved face masks revealed that the transmission efficiency rests with the choice of fabric, the number of layers in random or ordered arrangements, elastic response to repeated stretch or strain, and its recovery. Masks that can withstand a greater load under stretch are more durable and can be extended to last longer. These include *reusable cotton*, antiviral, and silk masks. The porosity measurements revealed the FFP2 and FFP3 masks have the greatest filtration efficiency. However, their life is limited to fewer wearing cycles in normal use due to the likelihood of permanent damage (FFP2, FFP3, and surgical masks). The porosity within the front layer of these masks ranges from 2.5 to 24%. Despite this, a comparable figure exceeding 95% of resistance to transmissibility is achieved by increasing the number of layers as necessary. Mechanical testing showed that for a larger fibre diameter with reduced strength, the disposable masks have the least pore size compared to reusable masks. However, half of the amount of work involved in extending repeatedly a disposable mask beyond its elastic limit results in irreversible damage to the fabric. Further studies on the filtration efficiency of a wider range of medical and non-medical face masks is continuing given the recent resurgent of the COVID virus.

## Data Availability

The datasets generated and/or analysed during the current study are available from the corresponding author on reasonable request.
